# From marmosets to man: generating a protocol for directly converted neurons to advance Alzheimer's Disease research

**DOI:** 10.1002/alz70855_107498

**Published:** 2025-12-25

**Authors:** Thais Rafael Guimarães, Jung Eun Park, Catrina Spruce, Stephanie Hachem, Swati Banerjee, Lauren K Hayrynen Schaeffer, Gregg E Homanics, Stacey J Sukoff Rizzo, Gregory W Carter, Afonso C Silva, Amantha Thathiah

**Affiliations:** ^1^ University of Pittsburgh School of Medicine, Pittsburgh, PA, USA; ^2^ The Jackson Laboratory, Bar Harbor, ME, USA

## Abstract

**Background:**

The progress of pre‐clinical research on Alzheimer's Disease (AD) has been hindered by the use of models that do not faithfully recapitulate human aging and frank AD neuropathology. The common marmoset (*Callithrix jacchus*) is a New World non‐human primate (NHP) with aging, genetic variability, and social behaviors that closely resemble those of humans, making it an invaluable model for studying age‐related disorders such as AD. Furthermore, research on marmosets overcomes a major limitation of human studies: the correlation of longitudinal *in vitro* models with *in vivo* assessments. Fibroblasts can be directly converted into induced neurons (iNs), bypassing pluripotent stages and thereby maintaining age‐associated phenotypes that are crucial for modeling the pathobiology of AD. However, while reproducible protocols for obtaining marmoset‐derived iNs are still in their early stages, they offer a tremendous opportunity to explore disease pathogenesis and identify therapeutic avenues for AD.

**Method:**

We utilized state‐of‐the‐art techniques to develop and optimize a protocol for directly converting marmoset fibroblasts into iNs. We characterized these marmoset‐derived iNs through various cellular assays, biochemical and imaging techniques, and RNA sequencing (RNAseq) and strengthened the validity of our protocol by direct comparison with human‐derived fibroblast to iN conversion.

**Result:**

We implemented a well‐validated human direct reprogramming protocol on marmoset fibroblasts. Surprisingly, marmoset fibroblasts do not survive the standard 21‐day human protocol for iN conversion. Consequently, we conducted an unbiased RNAseq study comparing the conversion trajectory of marmoset and human iNs, which revealed significant species‐specific differences. These differences guided modifications to our conversion strategies for marmoset iNs (e.g*.*, length of conversion, starting number of cells, media composition, frequency of media changes, substrate coating, pro‐survival supplementation, and gradual induction). Upon extensive optimization, we developed a robust, high‐efficiency protocol for marmoset iN conversion that preserved cell survival, enhanced neuronal maturity, and produced synaptically functional neurons in culture.

**Conclusion:**

Our novel protocol facilitates the study of cellular mechanisms with minimally invasive techniques, maximizing insights gained from marmoset models. This invaluable *in vitro* platform will also permit high‐throughput drug screening, safer toxicology assessment, and in‐depth mechanistic investigation of normal brain aging and AD pathogenesis, thus advancing translatable therapeutic opportunities for AD intervention.

